# Treatment of Actual Chemical Wastewater by a Heterogeneous Fenton Process Using Natural Pyrite

**DOI:** 10.3390/ijerph121113762

**Published:** 2015-10-28

**Authors:** Liang Sun, Yan Li, Aimin Li

**Affiliations:** State Key Laboratory of Pollution Control and Resources Reuse, School of the Environment, Nanjing University, Nanjing 210023, China; E-Mails: sunliangphd@tju.edu.cn (L.S.); liyan_0921@126.com (Y.L.)

**Keywords:** natural pyrite, heterogeneous Fenton process, chemical wastewater, reduction

## Abstract

Wastewater from chemical plants has remarkable antibiotic effects on the microorganisms in traditional biological treatment processes. An enhanced Fenton system catalyzed by natural pyrite was developed to degrade this kind of wastewater. Approximately 30% chemical oxygen demand (COD) was removed within 120 min when 50 mmol/L H_2_O_2_ and 10 g/L natural pyrite were used at initial pH from 1.8 to 7. A BOD_5_/COD enhancement efficiency of 210% and an acute biotoxicity removal efficiency of 84% were achieved. The COD removal efficiency was less sensitive to initial pH than was the classic Fenton process. Excessive amounts of pyrite and H_2_O_2_ did not negatively affect the pyrite Fenton system. The amount of aniline generated indicated that nitrobenzene reduction by pyrite was promoted using a low initial concentration of H_2_O_2_ (<5 mmol/L). Fluorescence excitation emission matrix analyses illustrated that H_2_O_2_ facilitated the reduction by natural pyrite of organic molecules containing an electron-withdrawing group to electron-donating group. Thus, the Fenton-like process catalyzed by pyrite can remediate wastewater containing organic pollutants under mild reaction conditions and provide an alternative environmentally friendly method by which to reuse natural pyrite.

## 1. Introduction

Wastewater from chemical plants is a significant source of environmental contamination [1,2,3]. This kind of wastewater has remarkable antibiotic effects on microorganisms in traditional biological treatment processes because various organic compounds in the wastewater, especially nitro-aromatic compounds such as nitrobenzene, are toxic and bio-refractory [4,5]. Therefore, technologies that improve the biodegradability of this type of wastewater should be developed.

Various methods have been proposed to address this challenge. Physical-chemical technologies have been used to modify the chemical characteristics of wastewaters and render them treatable in biological systems without adverse effects. Advanced oxidation processes, such as Fenton reactions [6,7], ozonation [8,9], and photochemical oxidation [10,11] have shown great potential due to their high efficiency in removing refractory compounds. These technologies can mineralize contaminants completely and have been intensively investigated. In particular, the Fenton reactions have been proven to be one of the best choices for practical application because of their high efficiency, simple operation, and low cost. However, in spite of the high oxidation performance, the classic Fenton reaction (which is catalyzed by soluble Fe^2+^) has some critical limitations: The operation needs to start under low initial pH (the optimum pH usually is 3) and the stoichiometric quantity of soluble Fe^2+^ that must be added generates significant amounts of sludge [12,13,14]. In the last few decades, the use of iron-bearing oxides (instead of soluble Fe^2+^) as heterogeneous catalysts has received increasing attention as a means by which to overcome these drawbacks. Several studies have reported that the use of iron-bearing oxides as catalysts in Fenton reactions has advantages of low cost and easy operation, and may exhibit excellent catalysis performance in the removal of organic contaminants [15,16,17].

Pyrite (FeS_2_) is the most abundant metal sulfide on the surface of Earth [18] and can be an appropriate material to act as a heterogeneous catalyst in the Fenton reaction. For example, Matta and Arienzo used a pyrite Fenton system for the oxidative degradation of 2,4,6-trinitrotoluene and reported that the observed degradation kinetics were much faster than those in the presence of other iron minerals such as magnetite and ferrihydrite [15,19]. Che and Bae used a pyrite Fenton system for the oxidative degradation of trichloroethylene and diclofenac, and reported that the degradation of both compounds was better in the pyrite Fenton system than in a classic Fenton system [20,21]. Wu found that hydrogen peroxide (H_2_O_2_) enhanced by natural pyrite had great activity in the decoloration of azo dyes [22]. Zhang demonstrated that the degradation of nitrobenzene in the pyrite Fenton system was significantly enhanced compared to that achieved in a classic Fenton system [23].

However, there is little in the literature describing the application of the pyrite Fenton process to treat actual wastewater. The performance of this technology in remediating chemical wastewater is not yet known. Data are needed that define the catalytic performance of the pyrite Fenton process for the treatment of chemical wastewater so that the feasibility and application of this technology can be evaluated.

Meanwhile, reducing the quantity of contaminants may not necessarily be effective in reducing health and environmental risks because some degradation products may be more toxic than their parent compounds [24]. Several examples of this case have been reported in wastewater treatment processes [2,24]. Thus, information on the biodegradability and biotoxicity of chemical wastewater treated using the pyrite Fenton process is essential to evaluating the ecological safety and overall feasibility of this technology.

In the present study, the heterogeneous Fenton process using natural pyrite has been developed. The reactivity performance of the pyrite Fenton system was compared to that of a classic Fenton system when given the same initial conditions (iron content, H_2_O_2_ concentration, initial pH), and the effects of the dosage of pyrite and H_2_O_2_ on the removal of chemical oxygen demand (COD) were evaluated in detail. The biodegradability and biotoxicity of the treated wastewater were also assessed. Fluorescence excitation emission matrix (EEM) analysis was used to characterize the change of functional groups in the wastewater before and after treatment using the pyrite Fenton process. The results showed that adding a small quantity of H_2_O_2_ could enhance the reducing performance of the natural pyrite. The results contribute to a better understanding of the mechanism and reaction process of the pyrite Fenton technology. This report is the first to describe the reduction of nitrobenzene through Fenton oxidation catalyzed by natural pyrite.

## 2. Experimental Section

### 2.1. Reagents and Wastewater

Chemicals used in the experiments consisted of reagent grade (AR) H_2_O_2_ (30%), iron (II) sulfate heptahydrate (FeSO_4_·7H_2_O), sulfuric acid (H_2_SO_4_, 98%), hydrochloric acid (HCl), and sodium hydroxide (NaOH) and were obtained from the Sinopharm Chemical Reagent Co. Ltd., Shanghai, China. All chemicals were used without further purification. The pyrite used in the experiments was mined from Anhui, China. The pyrite was sieved to a 300-mesh powder, washed with 1 mol HCl to remove surface oxidation layers, rinsed three times with deoxygenated deionized water and dehydrated with ethanol, and dried and stored in a closed vial under a pure nitrogen atmosphere. Elemental analyses showed that the iron content of the pyrite was 25%. The BET surface area of the pyrite was 5.960 m^2^/g.

The wastewater samples used in experiments were collected from an industrial chemical plant located in the Jiangsu province in southeast China. The plant engages in the production of chemical intermediates for pharmaceuticals, dyes, and pesticides. The main products are *P*-nitrotoluene (PNT) and *O*-nitrotoluene (ONT). The wastewater samples were taken from the nitration process and nitrobenzene was the major by-product during the process. The characteristics of the wastewater are given in Table 1.

**Table 1:** Water quality indexes of wastewater used in experiments.

Index	COD (mg/L)	BOD_5_/COD	TOC (mg/L)	Acute Biotoxicity (mg Zn^2+^/L)	Nitrobenzene (mg/L)	pH	Conductivity (μS/cm)
Values	7500–8000	0.1	2000	471–490	>300	1.8	38,000

### 2.2. Degradation of Chemical Wastewater by the Pyrite Fenton System and the Classic Fenton System

Experiments on the pyrite Fenton and classic Fenton systems were conducted in 250-mL Erlenmeyer flasks. Chemical wastewater (200 mL) and a dosage of natural pyrite (2 g) were mixed, which was calculated to a solid-liquid ratio of 10 g/L, yielding an iron (as Fe(II)) concentration of 2500 mg/L (44.64 mmol/L). The degradation process was initiated by adding 1.02 mL of H_2_O_2_ (30%) into the flask, yielding an initial concentration of 50 mmol/L H_2_O_2_, after which the resulting slurry was mixed using a mechanical stirrer (150 rpm) at 25 °C. The classic Fenton system was operated under the same initial experimental conditions as for the pyrite Fenton system except 44.64 mmol/L of FeSO_4_·7H_2_O was used as an aqueous iron source instead of pyrite. Experiments were conducted at various initial pH (1.8–7), obtained by adding diluted sulphuric acid (10%) or sodium hydroxide solution (5 mol/L). Samples (1 mL) of solution were retrieved from each reaction flask at regular intervals (every 20 min) for further analysis using the techniques described below under “Analytical methods”. Before the measurement of COD, BOD_5_, acute biotoxicity, and the concentration of nitrobenzene and aniline, the pH of samples were adjusted to 8–9 to remove the residual aqueous solution iron and then they were aerated with N_2_ for 30 min to remove the residual H_2_O_2_. The measurement of the concentration of Fe^2+^, total aqueous Fe did not adjust the values of pH of samples. All experiments were performed in duplicate.

### 2.3. Effects of the Dosage of Pyrite and H_2_O_2_

The effects of the dosage of pyrite and H_2_O_2_ were investigated in batch experiments. All experiments were performed in Erlenmeyer flasks at 25 °C. To observe the effects of pyrite and H_2_O_2_ dosages, the pyrite concentrations were set as 5, 10, 20, and 50 g/L, and the H_2_O_2_ concentrations were set at 1, 2, 5, 10, 50, and 100 mmol/L. All experiments were performed in duplicate.

### 2.4. Analytical Methods

The COD concentration was measured by COD analyzer (HACH, DRB 200, Loveland, CO, USA), respectively [25]. The BOD_5_ was measured by the amount of oxygen consumed by the decomposition of organic matter in wastewater over 5 days [25]. The BOD_5_/COD index (B/C) was used to assess the wastewater biodegradability.

The acute biotoxicity measurements were conducted using the widely used photobacterium bioassay. This method quantifies the decrease in light emission of the bioluminescent bacteria *Photobacterium phosphoreum*, which results from exposure to pollutants. The extent of luminescence inhibition after 5 min exposure is standardized into an equivalent concentration of Zn^2+^, which is used to express the degree of pollutant effects on the test bacteria. Thus, a greater luminescence inhibition corresponds to a higher equivalent Zn^2+^ concentration [26].

The concentration of Fe^2+^ was measured using to the 1,10-phenanthroline method [27]. The total aqueous Fe concentration was determined using flame atomic absorption spectrophotometry. The concentration of nitrobenzene was measured using the reduction azo-photometry [25], and the amount of aniline in the samples was determined using the *N*-(1-Naphthalene)-Ethylenediamine (*N*-(1-naphthalenyl)-1,2-ethanediamine; *N*-na; C_10_H_7_NHCH_2_CH_2_NH_2_) method according to previous research [28].

All samples with no dilution were used for EEM analysis with Shimadzu UV-1800 ultraviolet-visible (UV/vis) spectrophotometer. The EEM fluorescence spectra were obtained as follows. The scanning field was set at an excitation wavelength from 245 to 400 nm and the emission wavelength from 280 to 500 nm, with 5- and 1-nm sampling intervals in excitation (Ex) and emission (Em) modes, respectively. All fluorescence data were presented in arbitrary units. A PARAFAC analysis was used to interpret the EEM data after first completing several preparative steps (*i.e.*, data loading, scattering removal, and initial explorative data analysis) [29,30,31].

## 3. Results and Discussion

### 3.1. Comparison of COD Removal in the Pyrite Fenton System and the Classic Fenton System

**Figure 1:**
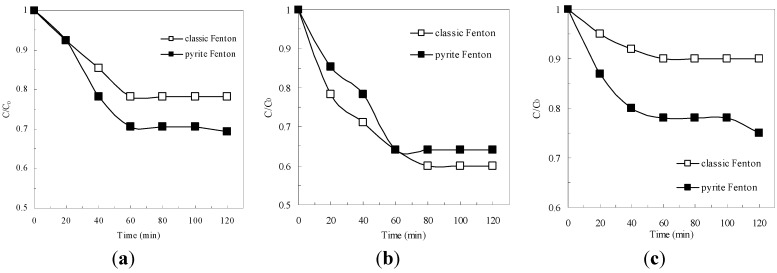
Comparison of pyrite Fenton and classic Fenton for the removal of COD under different initial pH. (**a**) initial pH = 1.8; (**b**) initial pH = 3; (**c**) initial pH = 7. Experimental conditions: (pyrite)_0_ = 10 g/L, (H_2_O_2_)_0_ = 50 mmol/L.

As shown in Figure 1a, at initial wastewater pH of 1.8, approximately 30% of the COD was removed in the pyrite Fenton system within 120 min, whereas only 20% of the COD was removed in the classic Fenton system in the same reaction time. Figure 1b shows that at the optimal initial pH for operation of the classic Fenton process (pH 3 as reported in previous papers [6]), the COD removal efficiency was greater than 30% in both Fenton systems (in 120 min), but the classic Fenton system removed more COD than did the pyrite Fenton system. At an initial pH of 7, the classic Fenton system did not degrade the organics of the wastewater (Figure 1c); in contrast, the COD removal efficiency was still greater than 25% in the pyrite-Fenton system.

These results demonstrated that pyrite has a benefit as Fenton reaction media because the initial pH had little influence on the removal of organics in the pyrite Fenton process. Previous authors reported that two types of oxidation occur during the pyrite Fenton process: pyrite oxidation (Equations (1)–(3)) and Fenton oxidation (Equations (4) and (5)) [21,22,23].

(1) $${\text{2FeS}}_{\text{2}}{\text{+7O}}_{\text{2}}{\text{+2H}}_{\text{2}}\text{O}\to {\text{2Fe}}^{\text{2+}}{{\text{+4SO}}_{\text{4}}}^{\text{2−}}{\text{+4H}}^{\text{+}}$$

(2) $${\text{FeS}}_{\text{2}}{\text{+14Fe}}^{\text{3+}}{\text{+8H}}_{\text{2}}\text{O}\to {\text{15Fe}}^{\text{2+}}{{\text{+2SO}}_{\text{4}}}^{\text{2−}}{\text{+16H}}^{\text{+}}$$

(3) $${\text{2FeS}}_{\text{2}}{\text{+15H}}_{\text{2}}{\text{O}}_{2}\to {\text{2Fe}}^{\text{3+}}{{\text{+4SO}}_{\text{4}}}^{\text{2−}}{\text{+2H}}^{\text{+}}{\text{+14H}}_{\text{2}}\text{O}$$

(4) $${\text{Fe}}^{\text{2+}}{\text{+H}}_{\text{2}}{\text{O}}_{2}\to {\text{Fe}}^{\text{3+}}\text{+HO}\cdot {\text{+OH}}^{\text{−}}$$

(5) $${\text{Fe}}^{\text{2+}}{\text{OH}}^{\text{+}}{\text{+H}}_{\text{2}}{\text{O}}_{2}\to {\text{Fe}}^{\text{3+}}{\text{OH}}^{\text{2+}}\text{+HO}\cdot {\text{+OH}}^{\text{−}}$$

**Figure 2:**
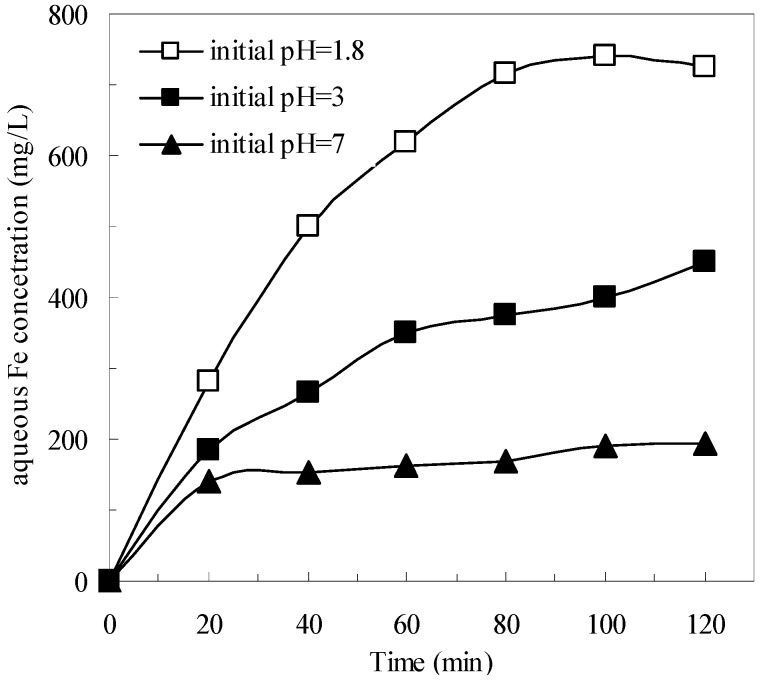
Aqueous Fe concentrations during the degradation in pyrite Fenton systems under different initial pH. Experimental conditions: (pyrite)_0_ = 10 g/L, and (H_2_O_2_)_0_ = 50 mmol/L.

Mechanisms on the formation of H_2_O_2_ and HO· by pyrite in oxic aqueous solutions were investigated systematically in previous studies [21,22,23]. In the presence of oxygen, pyrite can react to produce Fe^2+^ in the solution (Equation (1)), then the aqueous Fe^2+^ reacts with H_2_O_2_ to form HO· and changes to Fe^3+^ (Equation (4)). Therefore, in the pyrite Fenton system, both the generation of aqueous Fe^2+^ (Equation (1)) and of HO· (Equation (4)) could be major rate-limiting reactions that affect the removal of wastewater COD. However, in the classic Fenton system, the generation of HO· (Equation (4)) is the only rate-limiting reaction. As shown in Figure 2, the aqueous total Fe concentration in the pyrite system gradually increased, which indicated that the aqueous Fe^2+^ was generated gradually. When the process was applied at the initial wastewater pH of 1.8, the aqueous total Fe concentration was 725 mg/L after 120 min treatment. In contrast, the aqueous total Fe concentration was only 195 mg/L at an initial wastewater pH of 7, which resulted in less iron sludge being produced. In addition, the less iron concentration could be reduced to 21.2 mg/L after the subsequent coagulation and sedimentation process.

According to Equation (4), the initial pH plays a key role in the removal of organics in the classic Fenton system. To avoid the precipitation of Fe(OH)_3_, which consumes Fe^2+^ in the aqueous solution, the classic Fenton process is always operated at pH 3 (or lower) [32,33]. The present study demonstrated the validity of this conclusion. However, in the pyrite Fenton system, the COD removal efficiencies achieved at all initial pH values were quite similar, although the efficiency at pH 3 was somewhat superior to that at the other pH values. This phenomenon can be explained by the following reasons.

**Figure 3:**
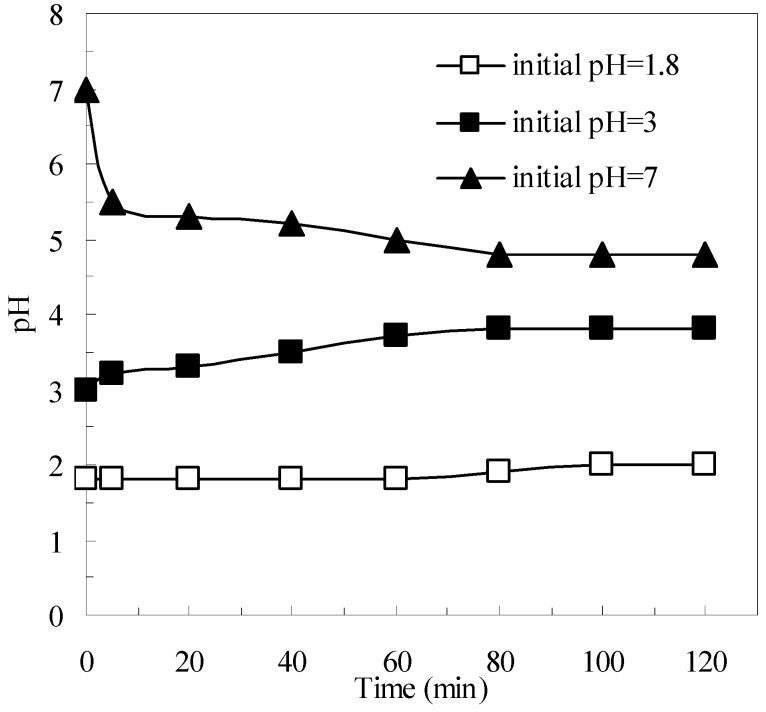
Variation of suspension pH with respect to time. Experimental conditions: (pyrite)_0_ = 10 g/L, and (H_2_O_2_)_0_ = 50 mmol/L.

As a heterogeneous material, pyrite has the primary advantage of helping to avoid the formation of iron oxide sludge [34]. Thus, pyrite can expand the effective pH range in which the Fenton process can operate successfully [21,23]. The variation of suspension pH with respect to time in the pyrite Fenton system is shown in Figure 3. As the reaction proceeded, the pH of the suspension underwent obvious changes. When the initial wastewater pH was extremely acidic, the pH slightly (within a few minutes) increased as the reaction progressed, then remained in the acidic range. In contrast, when the initial wastewater pH was at neutrality, the pH first decreased drastically from 7 to 5.5, and then gradually decreased to pH 4.8. As described by Equation (1), the pyrite can react with oxygen and generate protons, which is the reason that the pH decreased. Importantly, the decreased pH helped sustain the Fenton reaction. Therefore, in contrast to the classic Fenton system, the pyrite Fenton system can naturally achieve the appropriate pH conditions for effective Fenton reactions without the need for chemical additives.

### 3.2. Biodegradability Enhancement and Acute Biotoxicity Reduction by the Pyrite Fenton System

**Figure 4:**
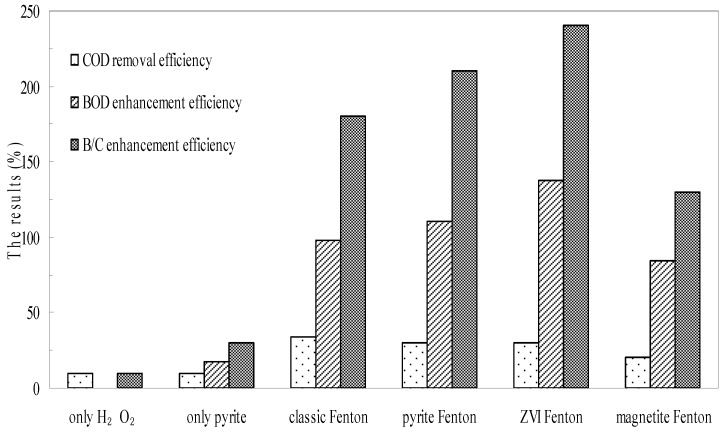
Comparison of pyrite Fenton and classic Fenton for the enhancement of B/C. Experimental conditions: (pyrite)_0_ = 10 g/L, (ZVI)_0_ = 10 g/L, (magnetite)_0_ = 10 g/L, (H_2_O_2_)_0_ = 50 mmol/L, and intial pH = 3.

To investigate the effect of biodegradability enhancement and acute biotoxicity reduction via different Fenton system, batch experiments using pyrite, zero valent iron, and magnetite were set up. The changes in the biodegradability of wastewater as a result of treatment are illustrated in Figure 4. The B/C index of the untreated chemical wastewater was nearly 0.1, which demonstrated that this kind of wastewater was resistant to treatment using traditional biological technologies. Neither oxidation by H_2_O_2_ or reduction by natural pyrite as individual processes improved the biodegradability of the wastewater remarkably. The B/C index following H_2_O_2_ oxidation was 0.11, and was 0.13 following pyrite reduction. However, after wastewater was treated using the Fenton processes, the B/C index increased to approximately 0.3 in the classic Fenton system, to 0.31 in the pyrite Fenton system, and to 0.34 in the ZVI Fenton system, while it was only 0.23 in the magnetite Fenton system. Thus, the pyrite Fenton system showed a remarkable ability to improve wastewater biodegradability.

As shown in Figure 4, the COD efficiency of oxidation by H_2_O_2_ was 10% within 120 min, however, the value of BOD was decreased, suggesting that the biodegradability enhancement was mainly caused by COD removal. In the pyrite system, the COD was removed less, but the BOD was increased remarkably. The result showed that pyrite has an extremely strong reducing capacity for pollutant removal. The electron-deficient groups of compounds that include nitrobenzene could be reduced to electron-donating groups (such as aniline) to increase the biodegradability of wastewater. Comparing the results of COD removal and biodegradability enhancement of pyrite Fenton system, the biodegradability data demonstrated more remarkable changes than COD removal. These results indicated that the organic compounds were not degraded completely but were transformed to other less toxic compounds. During the Fenton process, the macromolecular compounds were split to small molecule substances by HO·, and these reactions tend to simplify the structure of molecules to improve the biodegradability.

**Figure 5:**
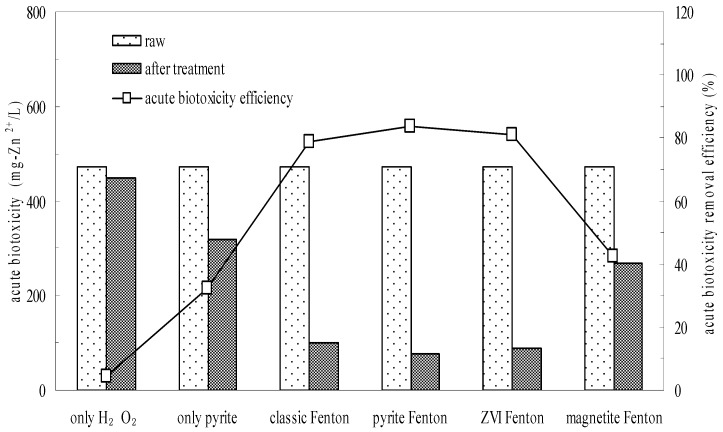
Comparison of pyrite Fenton and classic Fenton for the removal of acute biotoxicity. Experimental conditions: (pyrite)_0_ = 10 g/L, (ZVI)_0_ = 10 g/L, (magnetite)_0_ = 10 g/L, (H_2_O_2_)_0_ = 50 mmol/L, and intial pH = 3.

Batch experiments further confirmed the biotoxicity reduction effected by wastewater treatment. The acute biotoxicity values of the wastewater before and after treatment are shown in Figure 5. The initial overall toxicity of the influent wastewater was 471.4 mg Zn^2+^/L, which indicated that the wastewater posed a high environmental risk. As with their effects on wastewater biodegradability, neither H_2_O_2_ oxidation nor pyrite reduction acting alone reduced the acute biotoxicity of wastewater observably. The acute biotoxicity values of samples subjected to H_2_O_2_ oxidation and pyrite reduction were 450 and 320 mg Zn^2+^/L, respectively. However, the acute biotoxicity values of samples were remarkably reduced to 100, 77 and 90 mg Zn^2+^/L after the classic Fenton, pyrite Fenton and ZVI Fenton treatments, respectively. While it was 270 mg Zn^2+^/L in the magnetite Fenton system.

The results indicated that the magnetite Fenton system can not significantly improve the biodegradability and reduce the biotoxicity of chemical wastewater, while both the pyrite Fenton and ZVI Fenton technologies are the novel alternative to the classic Fenton process for simultaneously improving the biodegradability and reducing the biotoxicity.

### 3.3. Effects of the Pyrite and H_2_O_2_ on the Removal of Organics in the Pyrite Fenton System

**Figure 6:**
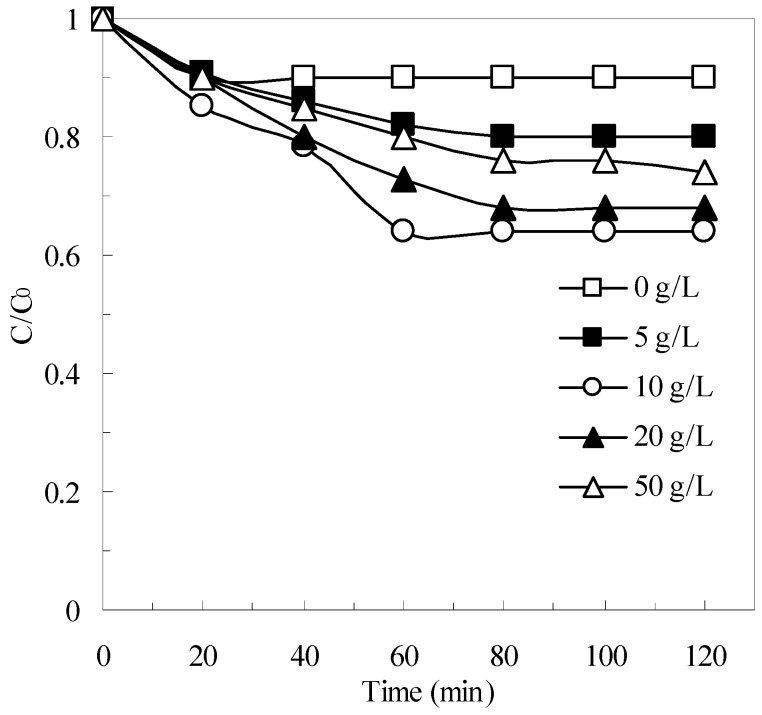
The effect of pyrite dosage on the removal of COD in pyrite Fenton system. Experimental conditions: (H_2_O_2_)_0_ = 50 mmol/L, and initial pH = 3.

The effect of natural pyrite dosage on the removal of pollutants was examined in batch experiments using different initial dosages of pyrite (0–50 g/L). The results are shown in Figure 6. Within 120 min, the COD removal efficiency reached 10%, 20%, 36%, 32%, and 26% at pyrite dosages of 0, 5, 10, 20, and 50 g/L, respectively. These results indicated that COD removal increased significantly as the initial pyrite concentration increased from 0 g/L to 10 g/L, whereas COD removal decreased slightly as the initial pyrite concentrations increased from 20 g/L to 50 g /L. Based on these results, a pyrite dosage of 10 g/L was optimal for the pyrite Fenton system treating the particular wastewater used in the experiments. This variation in COD removal as a function of pyrite dosage probably can be attributed to the follow reason.

According to Equations (1)–(3), increasing the amount of pyrite in suspension proportionally increases the aqueous Fe^2+^ concentration, which improves HO· formation (Equation (4)) and enhances the degradation of organics. However, previous authors pointed out that an excessive amount of aqueous Fe^2+^ in a pyrite suspension may promote the unwanted consumption of HO· (as shown in Equation (6)), which can negatively affect the oxidative degradation of COD by Fenton reactions [21,22,32].

(6) $${\text{Fe}}^{\text{2+}}\text{+HO}\cdot \to {\text{Fe}}^{\text{3+}}{\text{+OH}}^{\text{−}}$$

**Figure 7:**
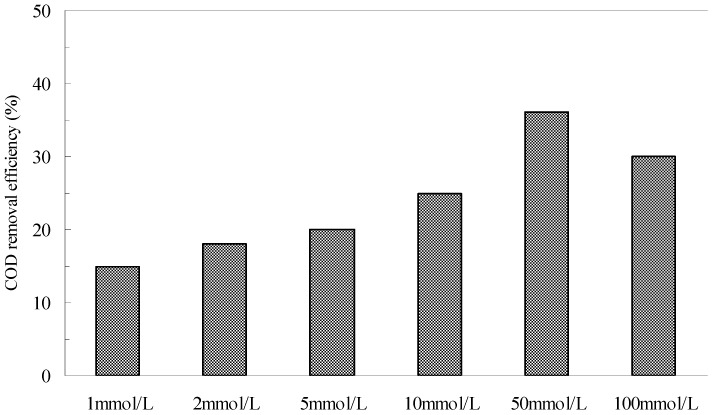
The effect of H_2_O_2_ concentrations on the removal of COD in pyrite Fenton system. Experimental conditions: (pyrite)_0_ = 10 g/L, and initial pH = 3.

Figure 7 shows the effect of the initial H_2_O_2_ concentrations on the removal of COD in the pyrite Fenton system. When the initial H_2_O_2_ was 1 mmol/L, the removal efficiency of COD was 15% after 120 min, but at an initial H_2_O_2_ concentration of 50 mmol/L, this efficiency increased to 36%. However, the TOC removal efficiency was only 10% under this condition. The results indicated that the organic compounds contained in the wastewater were not degraded completely but were split to small molecule substances by HO·. The stoichiometric amount of H_2_O_2_ for the complete mineralization of the carbon content to CO_2_ and H_2_O following Equation (7) was calculated to 33.33 mmol/L. It is clear that the actual dosage of H_2_O_2_ is more than the calculated value. Segura *et al.* also found this phenomenon in zero valent iron Fenton system [35]. They illustrated that the increase of the H_2_O_2_ showed a clear enhancement of performance when the highest oxidant loading (200% H_2_O_2_) was used. However, when the initial H_2_O_2_ increased to 100 mmol/L, the COD remove efficiency decreased to 30.

(7) $${\text{C+2H}}_{\text{2}}{\text{O}}_{\text{2}}\to {\text{CO}}_{\text{2}}{\text{+2H}}_{\text{2}}\text{O}$$

These variations were consistent with those observed in previous studies [6,36], which proposed that when an appropriate concentration of Fe^2+^ was provided in the reaction system, an increase of the H_2_O_2_ concentration could enhance the oxidative degradation of organics in the pyrite Fenton system due to the improvement in HO· formation. In contrast, an excessive amount of H_2_O_2_ in a pyrite suspension readily reacts with generated HO· (Equation (8)), reducing the oxidative removal of COD by HO·.

(8) $${\text{H}}_{2}{\text{O}}_{2}\text{+HO}\cdot \to \text{OOH}\cdot {\text{+H}}_{\text{2}}\text{O}$$

However, neither of the potentially negative effects posed by excessive amounts of pyrite and H_2_O_2_ appeared to be significant in the pyrite Fenton system. Relative insensitivity to such effects was another advantage that the pyrite Fenton system offered over the classic Fenton system.

### 3.4. The Generation of Aniline in the Pyrite Fenton System

**Figure 8:**
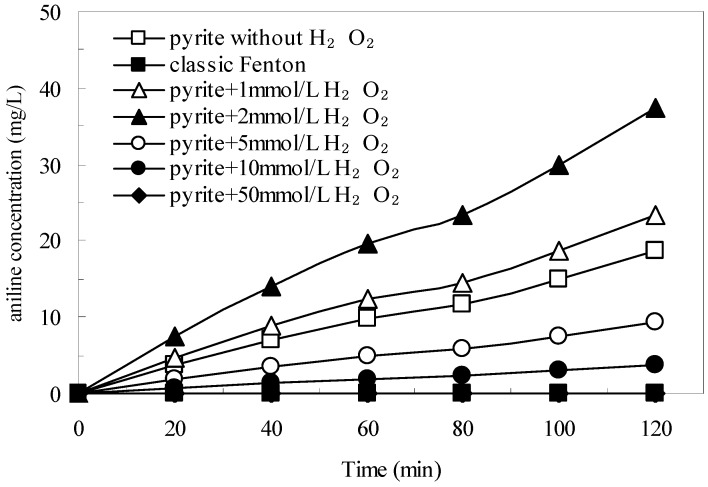
Amount of aniline in the aqueous solution with different initial H_2_O_2_ concentration. Experimental conditions: (pyrite)_0_ = 10 g/L, and initial pH = 1.8.

Figure 8 shows the influence of initial H_2_O_2_ concentration on the generation of aniline. The untreated wastewater did not contain aniline, and no aniline was generated in the classic Fenton process. In the pyrite Fenton system, when only pyrite was added, the concentration of aniline in the treated wastewater was 18.7 mg/L after 120 min, which indicated that the natural pyrite could have a reduction capacity for nitro-aromatic compounds. In response to H_2_O_2_, within 120 min the concentration of aniline reached 23.4, 37.5, 9.4, and 3.7 mg/L at initial H_2_O_2_ concentrations of 1, 2, 5, and 10 mmol/L, respectively. However, when the initial H_2_O_2_ concentration was 50 mmol/L, no aniline was detected in the aqueous solution. These results showed that low initial concentrations of H_2_O_2_ (from 1 mmol/L to 2 mmol/L) significantly improved the reduction performance of pyrite, whereas high concentrations of H_2_O_2_ (5 mmol/L to 50 mmol/L) inhibited the reduction performance.

Stumm and James proposed that the dissociation of pyrite releases dissolved ferrous and sulfur (S_2_^2−^) ions, which have an extremely strong reducing capacity for pollutant removal (Equation (9)) [37]. Zhang also found that the released dissolved ferrous effectively made pyrite an electron donor that resulted in the formation of Fe^3+^, whereas the nitro group of compounds that include nitrobenzene were electron-deficient and thus, they could be readily reduced [34].

(9) $${\text{FeS}}_{\text{2}}\to {{\text{Fe}}_{\text{(aq)}}}^{\text{2+}}{{\text{+S}}_{\text{2(aq)}}}^{\text{2−}}$$

Many studies have suggested that the decomposition of H_2_O_2_ can be controlled by surface-catalyzed process in the heterogeneous reaction involving iron oxides (e.g., magnetite, goethite, and hematite) [38,39]. Kong pointed out that the oxygen and H_2_O_2_ in an aqueous solution could oxidize the surface Fe(II) of pyrite to Fe(III), as described in Equations (10) and (11) [40]. Kwan found that most organics in the heterogeneous Fenton process were degraded by HO· produced from dissolved Fe^2+^ (Equation (4)), not by a surface-catalyzed process (Equation (10)) [41]. Bae also observed this phenomenon in the pyrite Fenton system and furthermore demonstrated that H_2_O_2_ could reduce the surface Fe(III) of pyrite to Fe(II), and that the regenerated Fe(II) could be released into the aqueous solution and react with H_2_O_2_ to produce HO· (Equation (4)), which could react with target contaminants [21].

(10) $${{\text{Fe}}_{\text{pyrite}}}^{\text{II}}{\text{+O}}_{\text{2}}\to {{\text{Fe}}_{\text{pyrite}}}^{\text{III}}{{\text{+O}}_{\text{2}}}^{\text{−}}$$

(11) $${{\text{Fe}}_{\text{pyrite}}}^{\text{II}}{\text{+H}}_{\text{2}}{\text{O}}_{2}\to {{\text{Fe}}_{\text{pyrite}}}^{\text{III}}\text{+HO}\cdot {\text{+OH}}^{\text{−}}$$

(12) $${{\text{Fe}}_{\text{pyrite}}}^{\text{III}}{\text{+H}}_{\text{2}}{\text{O}}_{2}\to {{\text{Fe}}_{\text{pyrite}}}^{\text{II}}\text{+OOH}\cdot {\text{+H}}^{\text{+}}$$

**Figure 9:**
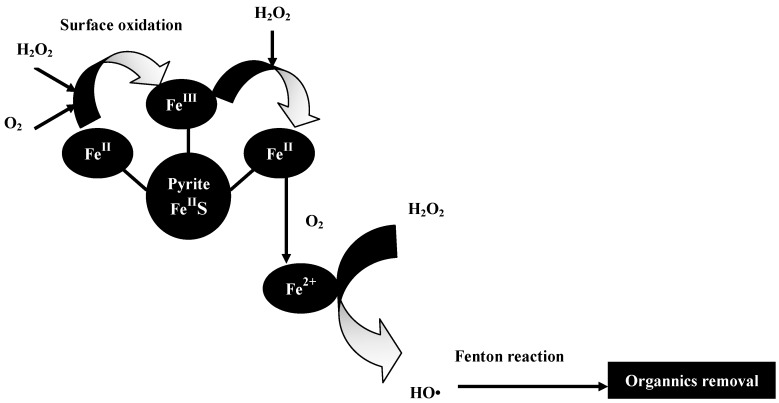
Proposed pathway for oxidative degradation of organics by pyrite Fenton system.

The pathway of this reaction process is shown in Figure 9. According to Equation (12), H_2_O_2_ can reduce the surface Fe(III) of pyrite to Fe(II) and enhance the generation of aqueous Fe^2+^. When the initial concentration of H_2_O_2_ is low (such as 1 mmol/L and 2 mmol/L), the residual H_2_O_2_ in the aqueous solution cannot oxidize the aqueous Fe^2+^ thoroughly; hence, there is ample aqueous Fe^2+^ to join in the reduction of nitrobenzene compounds to aniline. In the present study, aqueous Fe^2+^ concentrations reached 198 and 187 mg/L in the pyrite Fenton system (after 120 min) at initial H_2_O_2_ concentrations of 1 and 2 mmol/L, respectively. However, when the initial H_2_O_2_ concentration was sufficient (greater than 10 mmol/L), the aqueous Fe^2+^ was oxidized by the excess H_2_O_2_, indicating that Fenton oxidization is the main reaction that occurs during the entire process. In fact, the mechanism and entire process of the pyrite Fenton technology is complex and not very explicit. Further investigation of the reductive transformations and oxidative pollutant degradation that occurs in the pyrite Fenton system is ongoing.

### 3.5. Evaluation of Organic Functional Groups by EEM in the Pyrite Fenton System

EEM has been frequently used to characterize organics in water and wastewater treatment systems due to its high sensitivity, good selectivity, and non-destructive effect on samples [42,43]. As a technique of multivariate data analysis, PARAFAC can mathematically decompose the complex fluorescence spectra into individual fluorescent components for both quantitative and qualitative analysis [44,45]. In the present study, the EEM-PARAFAC analysis method was used to evaluate the organic functional groups in the wastewater before and after it was subjected to classic Fenton and pyrite Fenton treatment.

**Figure 10:**
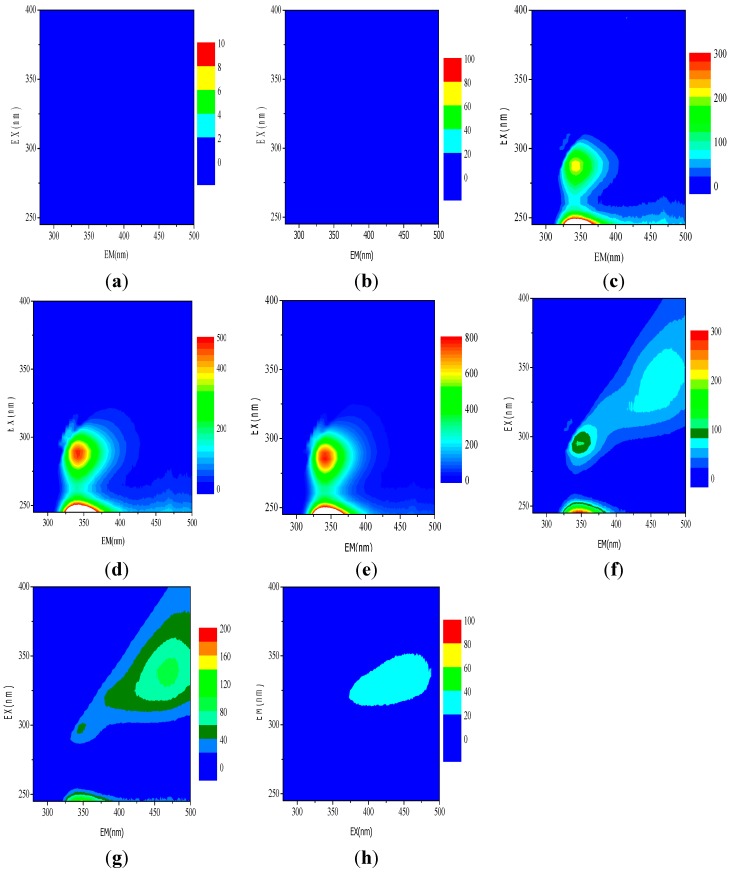
Sample results scanned by three-dimensional fluorescence. (**a**) raw water; (**b**) classic Fenton; (**c**) pyrite without H_2_O_2_; (**d**) pyrite + 1 mmol/L H_2_O_2_; (**e**) pyrite + 2 mmol/L H_2_O_2_; (**f**) pyrite + 5 mmol/L H_2_O_2_; (**g**) pyrite + 10 mmol/L H_2_O_2_; (**h**) pyrite + 50 mmol/L H_2_O_2_.

Figure 10 shows the three-dimensional fluorescence spectra for various samples. Using the peak-picking function of the instrument’s FL solution software, no peaks were found in the EEM spectra from either the raw (*i.e.*, untreated) wastewater or from wastewater treated using the classic Fenton process (Figure 10a,b); this indicated that there were no electron-donating groups contained in these samples. One major peak was found in the spectra from a number of samples at approximately Ex 290 nm and Em 432 nm (Figure 10c–g). The relative heights of the peaks for each sample were calculated to 211.1, 471.9, 772.2, 102.3, and 41.87, respectively. No spectral peak was observed for the sample that included pyrite and 50 mmol/L H_2_O_2_ (Figure 10h). Generally, fluorescent peaks at Ex 290 nm and Em 432 nm originate from amine groups (-NH_2_), and the height of a peak corresponds with the quantity of organic material that contains amine groups (such as aniline) [46]. The spectral responses shown in Figure 10 indicated that low initial H_2_O_2_ concentrations facilitated the reduction of organic molecules containing electron-withdrawing groups (such as -NO_2_) to electron-donating groups (such as -NH_2_) by pyrite. These results proposed that increased H_2_O_2_ concentration could enhance the reduction of nitrobenzene in a pyrite Fenton system by improving the surface Fe(II) regeneration.

## 4. Conclusions

In this research, the treatment of actual chemical wastewater using an enhanced Fenton system catalyzed by natural pyrite was evaluated systematically. The results shows that the Fenton process catalyzed using natural pyrite effectively removes COD across a broad range of initial pH (pH 1.8 to 7). Furthermore, the pyrite Fenton process effectively increases the biodegradability of treated wastewater and simultaneously reduces the toxicity of treated wastewater. Compared to that of the classic Fenton process, the COD removal efficiency of the pyrite Fenton process is less sensitive to the initial solution pH and relatively insensitive to excessive amounts of both pyrite and H_2_O_2_. Finally, in the pyrite Fenton system, the reduction of nitrobenzene and organic molecules containing electron-withdrawing groups to electron-donating groups by natural pyrite can be promoted by adding a small amount of H_2_O_2_ (initial concentration <5 mmol/L). Thus, the pyrite Fenton process offers several operational advantages over the classic Fenton process and is a viable method by which to remediate organic pollutants in wastewater, while providing an environmentally friendly alternative for reusing natural pyrite.
